# Biannual Spawning and Temporal Reproductive Isolation in *Acropora* Corals

**DOI:** 10.1371/journal.pone.0150916

**Published:** 2016-03-10

**Authors:** James P. Gilmour, Jim N. Underwood, Emily J. Howells, Emily Gates, Andrew J. Heyward

**Affiliations:** 1 Australian Institute of Marine Science (AIMS), The University of Western Australia Oceans Institute, Perth, Western Australia, Australia; 2 Australian Institute of Marine Science (AIMS), Townsville, Queensland, Australia; University of Massachusetts, UNITED STATES

## Abstract

Coral spawning on the oceanic reef systems of north-western Australia was recently discovered during autumn and spring, but the degree to which species and particularly colonies participated in one or both of these spawnings was unknown. At the largest of the oceanic reef systems, the participation by colonies in the two discrete spawning events was investigated over three years in 13 species of *Acropora* corals (*n* = 1,855 colonies). Seven species spawned during both seasons; five only in autumn and one only in spring. The majority of tagged colonies (*n* = 218) spawned once a year in the same season, but five colonies from three species spawned during spring and autumn during a single year. Reproductive seasonality was not influenced by spatial variation in habitat conditions, or by *Symbiodinium* partners in the biannual spawner *Acropora tenuis*. Colonies of *A*. *tenuis* spawning during different seasons separated into two distinct yet cryptic groups, in a bayesian clustering analysis based on multiple microsatellite markers. These groups were associated with a major genetic divergence (*G”*_*ST*_ = 0.469), despite evidence of mixed ancestry in a small proportion of individuals. Our results confirm that temporal reproductive isolation is a common feature of *Acropora* populations at Scott Reef and indicate that spawning season is a genetically determined trait in at least *A*. *tenuis*. This reproductive isolation may be punctuated occasionally by interbreeding between genetic groups following favourable environmental conditions, when autumn spawners undergo a second annual gametogenic cycle and spawn during spring.

## Introduction

Most tropical corals are simultaneous hermaphrodites that reproduce by broadcast spawning, releasing their eggs and sperm into the water column where fertilisation and larval development occur [1, 2]. Although this single mode of reproduction dominates, the timing and synchrony of spawning vary considerably among reefs according to the complex combination of proximate and ultimate drivers [3]. On the Great Barrier Reef, spawning is synchronised among many colonies and species over a few nights each year [4, 5] whereas on other reefs a similar proportion of species and colonies spawn over several months a year [6–8]. In most instances, however, reefs fall along a continuum between these extremes, but with seasonal peaks of spawning activity (e.g. [1, 2, 9–12]). Variation in spawning synchrony is a key mechanism for reproductive isolation in corals and influences the degree of interbreeding. Conspecific colonies at the same location must release gametes within a few hours of each other to ensure successful fertilisation [13–15], whereas temporal reproductive isolation occurs when spawning times on a reef differ by several hours to several months [16, 17].

Temporal reproductive isolation in conspecific colonies may be due to a split-spawning [18], when colonies do not mature around a single lunar cycle and spawning occurs over two consecutive months [1], or the protracted spawning period that occurs to varying degrees over consecutive nights, weeks and/or months on all reefs. In these instances, colonies may still interbreed during years where spawning is not split, or if colonies periodically spawn on the same nights within a protracted spawning period. However, spawning by conspecific colonies can also occur during discrete seasons separated by several months, such as on reefs in Kenya [19], Taiwan [20], Singapore [9], Papua New Guinea [3], the Great Barrier Reef [17, 21] and Western Australia [22, 23]. In these instances, colonies can interbreed only if they alternate their season of spawning or spawn during both seasons. The degree of reproductive isolation therefore depends on the extent to which cycles of gametogenesis are driven by environmental cues and energy reserves, or are genetically determined.

Variation in the time of coral spawning has been attributed to environmental cues such as solar insolation [24, 25], wind speeds [11], water temperature, lunar and tidal cycles [1, 9, 14, 26]. However, it is not known whether such cues vary substantially enough within a reef system to promote spawning by conspecific colonies during discrete seasons. Variation in environmental conditions and photosymbiont type also influence colony energy reserves [27], and potentially cycles of gametogenesis. As gametogenesis requires a considerable energy investment [28, 29], most coral colonies spawn only once a year. However, biannual spawning by colonies during discrete seasons can occur [3, 8, 21, 30], suggesting its potential under optimal conditions. Few studies have followed patterns of reproduction in individual corals across seasons and years, or investigated the causes of variation in times of spawning. If spawning seasonality is a fixed genetic trait, conspecific subpopulations that spawn in different seasons will not interbreed over many generations, which may ultimately lead to reproductive isolation, divergence and subsequent speciation.

In this study, we investigated cycles of reproduction in biannually spawning corals on an oceanic reef system off Western Australia, and whether spawning seasonality was associated with environmental or genetic variation. Over three years, we surveyed the participation of *Acropora* assemblages in a primary mass spawning in autumn and a secondary spawning in spring. Our design included re-sampling individual colonies to determine their capacity to spawn twice a year or in different seasons. Local environmental drivers of reproductive cycles were investigated by sampling from sites across the reef system (separated by m to 10s km) and by identifying the *Symbiodinium* type hosted by colonies of the most abundant biannual spawner, *Acropora tenuis*. Additionally, genetic variation was quantified for colonies of *A*. *tenuis* from the sampling sites across the reef system to determine the extent of differentiation among sites versus colonies spawning during different seasons.

## Materials and Methods

### Study sites and environmental conditions

The study was conducted at North and South Scott Reef, located approximately 270km from the mainland of north-western Australia (S14° 04’, E121° 46’), where *Acropora* species typically contribute around 25% of the total coral cover [31]. Five study sites with contrasting environmental conditions and separated by four to 32 km were selected from across the reef system. Physical conditions were quantified during the study period (S1 Text) and the parameters that best explained the variation among sites were summarised (S1 Fig). The parameters that best distinguished site conditions were: percentage cover of sand, cumulative wind speeds, range in water temperature, rate and composition of sedimentation, turbidity and chlorophyll concentration (fluorescence), and maximum current speed and wave height. For all variable parameters, daily averages were calculated and divided between summer and winter months, to account for the influence of monsoonal conditions.

### Coral reproductive surveys

Reproduction was investigated in 13 abundant species of broadcast spawning *Acropora*: *A*. *cytherea*, *A*.*digitifera*, *A*. *florida*, *A*. *gemmifera*, *A*. *humilis*, *A*. *hyacinthus*, *A*. *microclados*, *A*. *millepora*, *A*. *muricata*, *A*. *polystoma*, *A*. *samoensis*, *A*. *spicifera* and *A*.*tenuis*. Coral colonies typical of the species morphology were targeted and all species identifications were confirmed *in situ* and with skeletal type specimens by the authors and by D. McKinney [32, 33], with reference types checked with the Museum of Tropical North Queensland. Of the 244 colonies initially tagged and followed through time, 26 were excluded from the study because of confusion in their species identity. To complement the genetic analysis (below) of the dominant biannual spawner (*Acropora tenuis*), an additional 25 colonies were collected for detailed skeletal analyses by Z. Richards from the Western Australia Museum, which included colonies known to spawn in autumn (*n* = 16) and spring (*n* = 9).

Mature (> 20 cm diameter) colonies (*n* = 50 to 416 colonies per species) were sampled randomly one to five weeks before the spawning in autumn and spring, in 2008, 2009 and 2010. In addition, 218 tagged colonies (*n* = 11 to 33 per species) were re-sampled prior to each spawning. Three branches were sampled from the central region of each colony to provide an *in situ* rank of egg development and spawning time based on the size and pigmentation of visible eggs: Score 1, indicated that colonies would spawn within the month; Score 2, indicated colonies would spawn within two months; Score 3, indicated that colonies had recently spawned, or will not spawn for several months. A subset of random colonies was checked *in situ* after the autumn spawning each year to confirm that mature eggs had been spawned. Sampling was approved by the Western Australian Department of Fisheries under the permit numbers 2008–6 and 14–795.

The branches from all colonies were fixed in 10% formalin-seawater, decalcified in 5–10% HCl, and stored in 70% ethanol for further analyses in the laboratory. Five polyps were randomly selected and dissected from the middle or base of each of three branches from each colony, and all eggs measured and counted. The geometric mean for each egg was calculated as the square root of the maximal x medial diameter [34]. If no eggs were present, a further 10 polyps were checked.

### Genetic analysis of *A*. *tenuis*

Tissue samples were collected from the tagged *A*. *tenuis* colonies sampled for reproductive analysis in 2010, to determine whether colonies that spawned in autumn or spring belonged to genetically distinct groups. *Acropora tenuis* was chosen for the genetic analysis because it is among the most common and easily distinguishable of the all biannual spawning corals. Fifty-two colonies were collected from the five sites used in the environmental analyses spread across North and South Scott Reef, and were assigned as either autumn (*n* = 25) or spring (*n* = 24) spawners, or as spawning in both seasons (*n* = 3) according to the field and laboratory observations. Total genomic DNA was extracted and allelic variation measured at five microsatellite loci developed for closely related species which are also polymorphic in *A*. *tenuis*: *Amil2_10*, *Amil2_11*, *Amil_12*, *Amil_018* [35] and *Acr_53* [36]. Microsatellites were amplified from genomic DNA in singleplexed polymerase chain reactions (PCR) at an annealing temperature of 50°C using fluorescently labelled primers. PCRs were performed in 10μl volumes using MyTaq reagents (Bioline) at a final concentration of 1 x buffer, 0.5 μM each primer, and 0.25 units of Taq. PCR products were purified with Sephadex (GE Healthcare), and genotyped by capillary electrophoresis (MegaBACE 1000, Genetic Analysis Facility, James Cook University). Fluorescence peaks at each locus were scored using MegaBACE Fragment Profiler 1.2 (Amersham Biosciences).

Population genetic statitistics including shared multilocus genotypes and genotype probability (GP), number of alleles, the expected heterozygosity, observed heterozygosity and fixation index at each locus within reproductive season (as defined by the reproductive survey) were calculated with GenAlEx v6 [37]. Shared multilocus genotypes were deemed to be clonal (produced by vegetative fragmentation) if they were collected from the same site and had a GP < 0.001.

Genetic divergence among *A*. *tenuis* colonies that spawned in different reproductive seasons was investigated with a Bayesian clustering analysis in STRUCTURE v2.3 [38] in two stages. An initial analysis was run to identify the optimal number of genetic clusters, *K*, in the entire sample without any prior information on spawning time of individual colonies. Mean and variance of log likelihoods and posterior probabilities of the number of clusters (*K* = 1 to 6) were inferred from multilocus genotypes by running STRUCTURE ten times using the admixture and independent allele frequencies models (burn in period of 100,000 and MCMC repetitions after burn-in of 1,000,000). The independent allele frequency model was implemented because the genetic differentiation between spawning groups was large, and trial runs with correlated allele frequencies model overestimated *K* in which individuals lacked strong assignments (see [39]). Convergence of algorithm was checked by assessing the stability of runtime α and Ln likelihood after burn in and the variability in individual assignment proportions across runs. The online program Clumpak [40] was used to summarise and graphically represent the STRUCTURE results from the ten independent runs as well as assess the true number of clusters using the method of Evanno *et*. *al*. [41].

The initial analysis unambiguously identified two distinct clusters, with the majority of individuals assigned to their correct spawning season (as identified by the reproduction survey). A secondary clustering analysis was implemented for *K* = 2 which utilised information on spawning season from the reproductive survey to assist clustering with the LOCPRIOR model. This model places prior weight on clustering outcomes that are correlated with phenotypic characteristics (in this case spawning season) and is recommended when a small number of markers and individuals are employed [42]. For this analysis, the three individuals that spawned in both seasons were pre-defined as a third group, and thus did not have prior information that assisted clustering into the autumn or spring clusters. In this way, we could assess the proportion of ancestry of these individuals to each cluster. All other parameters were the same as in the initial clustering analysis.

The level of differentiation between genetic groups identified by STRUCTURE was measured with *F*_ST_ and with *G”*_ST_ [43] in GenAlEx v6 [37] and excluded the three corals that spawned in both seasons. *G”*_ST_ accounts for the high degree of variation within populations, and is the recommended measure of genetic differentiation for inferences of demographic history, particularly when microsatellite markers are used [43].

### Genetic analysis of *Symbiodinium* in A. *tenuis*

*Symbiodinium* type(s) were genotyped from the *A*. *tenuis* genomic DNA used in the microsatellite analysis to determine any variation in photosymbiont communities among colonies spawning in different seasons. PCR was used to amplify the *Symbiodinium* ITS2 rDNA region using the primers *ITSintfor2* and *ITS2clamp* [44] using the protocol of LaJeunesse [45]. PCRs were performed in 20μl volumes using MyTaq reagents (Bioline) at a final concentration of 1 x buffer, 0.5 μM each primer, and 0.25 units of Taq. A touch-down protocol was used where the initial annealing temp of 62° was decreased by 0.5° each cycle until the target annealing temp of 52° was reached, with a final extension of 30 minutes PCR products were separated with denaturing gradient gel electrophoresis (DGGE) as described in [46]. DGGE banding profiles were the same among samples, and representative dominant bands plus faint bands were isolated for sequencing with a total of 3 replicates of each. These isolated bands were used in PCR with primers *ITSintfor2* and *ITSReverse* (without the GC clamp), and re-run on a DGGE and a subset of samples in which bands that matched the original DGGE profiles were sent for sequencing in both directions (Macrogen, Korea). Automated sequencing calls of 281 bases were visually checked and consensus sequences were aligned (Sequencher, Gene Codes Corporation) and compared to available *Symbiodinium* ITS2 rDNA sequences downloaded from GenBank (http://www.ncbi.nlm.nih.gov/genbank/).

## Results

### Spawning patterns in *Acropora* assemblages

**Fig 1:**
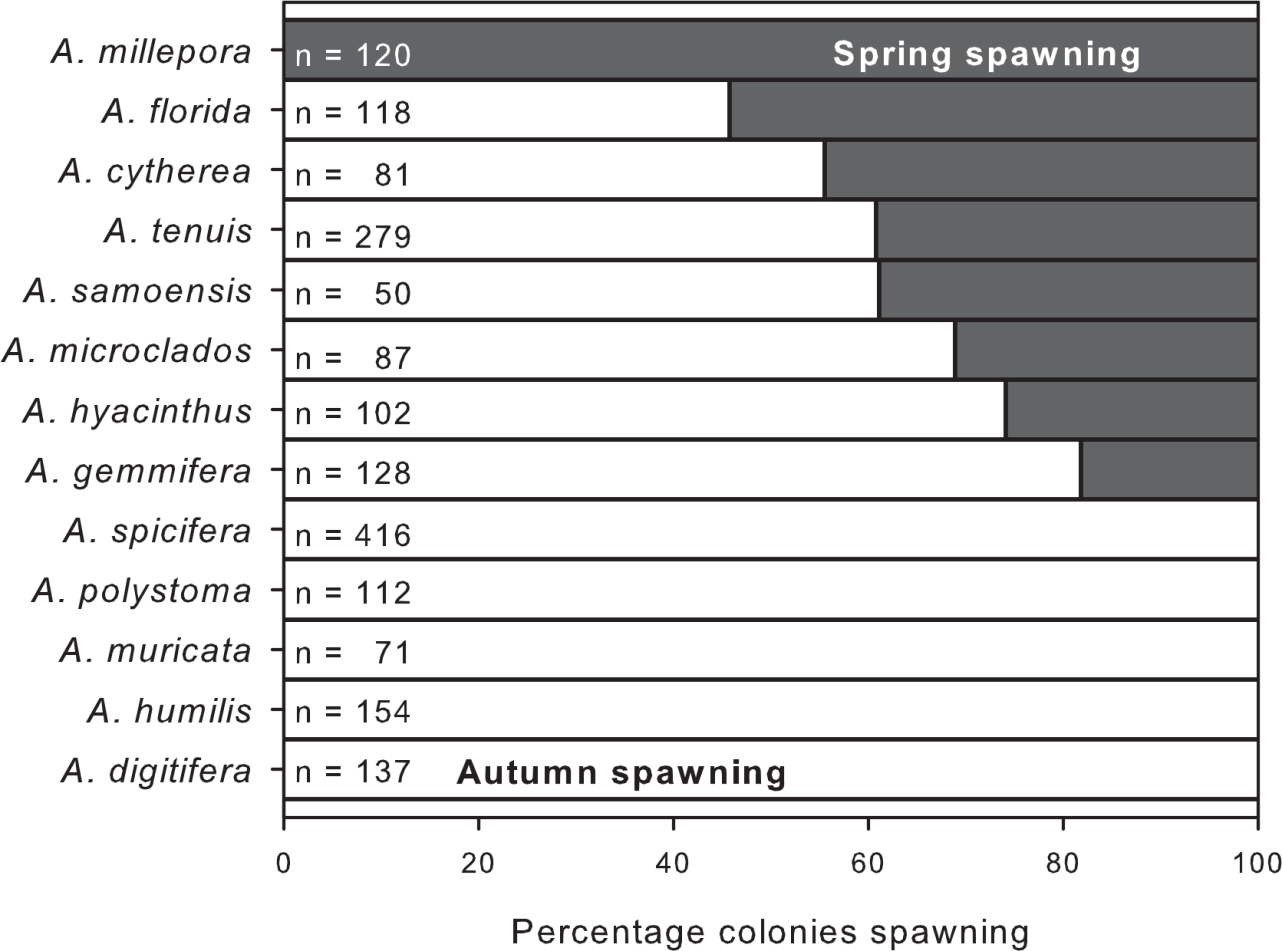
Biannual spawning in *Acropora* assemblages. Percentage of colonies within each species spawning during autumn (white bars) and/or spring (black bars). Colonies were sampled prior to the predicted dates of spawning in each season from 2008 to 2010.

**Fig 2:**
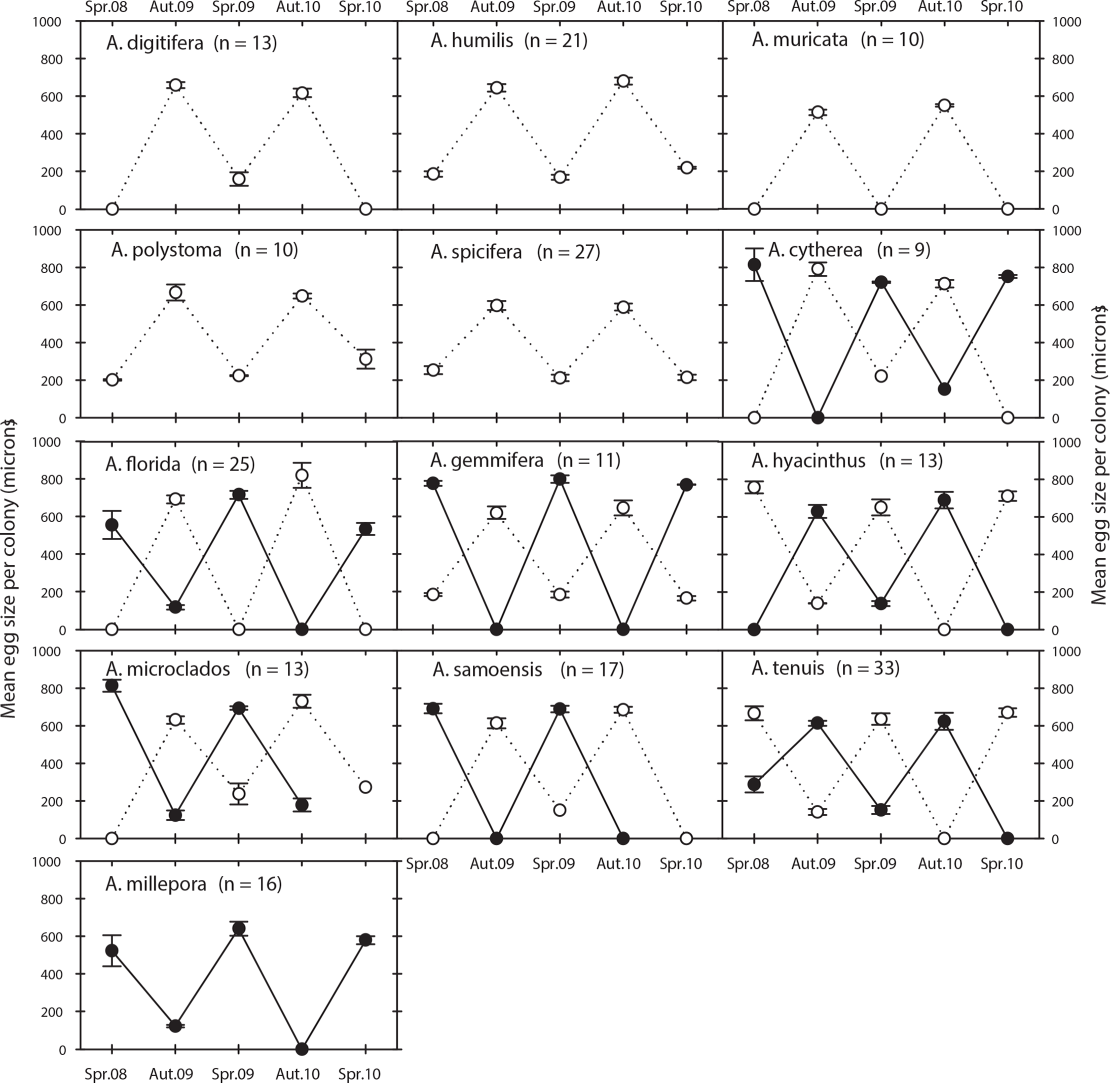
Individual oogenic cycles within *Acropora* assemblages. Mean (± S.E.) egg sizes for colonies re-sampled prior to the spawning in spring (Spr.) and autumn (Aut.) from 2008 to 2010. Within species, colonies spawned during spring (black circle and solid line) and/or autumn (white circle and dashed line). All colonies spawned only once a year during the same season, with the exception of five colonies of three species that spawned twice during a single year. A mean egg size of zero indicates the absence of eggs from all colonies at the time of sampling.

Of the 13 species of *Acropora* sampled, one spawned only in spring (*A*. *millepora*), five spawned only in autumn (*A*. *digitifera*, *A*. *humilis*, *A*. *muricata*, *A*. *polystoma*, *A*. *spicifera*) and seven spawned in both seasons (*A*. *cytherea*, *A*. *florida*, *A*. *gemmifera*, *A*. *hyacinthus*, *A*. *microclados*, *A*. *samoensis*, *A*. *tenuis*) (Fig 1). Most (98%) of the 218 tagged colonies re-sampled over 3 years spawned only once a year and always during the same season. The exceptions were 5 colonies that apparently spawned over two consecutive seasons. Colonies of *A*. *tenuis* (3), *A*. *cytherea* (1) and *A*. *florida* (1) contained mature gametes in spring 2008 and again in autumn 2009, but in subsequent years all spawned only during autumn. For most (6 of 7) species of biannual spawners, a similar proportion of colonies spawned in each season. Eggs were present in 29% of the colonies in spring and 51% in autumn, and were either large (450–915 μm; mean = 656 μm) and pigmented, indicating they would be spawned within weeks, or small (100–270 μm; mean = 180 μm) and unpigmented, indicating they would mature in the next spawning season (Fig 2). Within all the re-sampled colonies, small immature eggs present during one spawning season were observed as large and mature eggs in the next spawning season. There was no evidence of spawning outside autumn and spring based on *in situ* observations and gamete development.

### Spawning seasonality, environmental variation and *Symbiodinium* types

**Fig 3:**
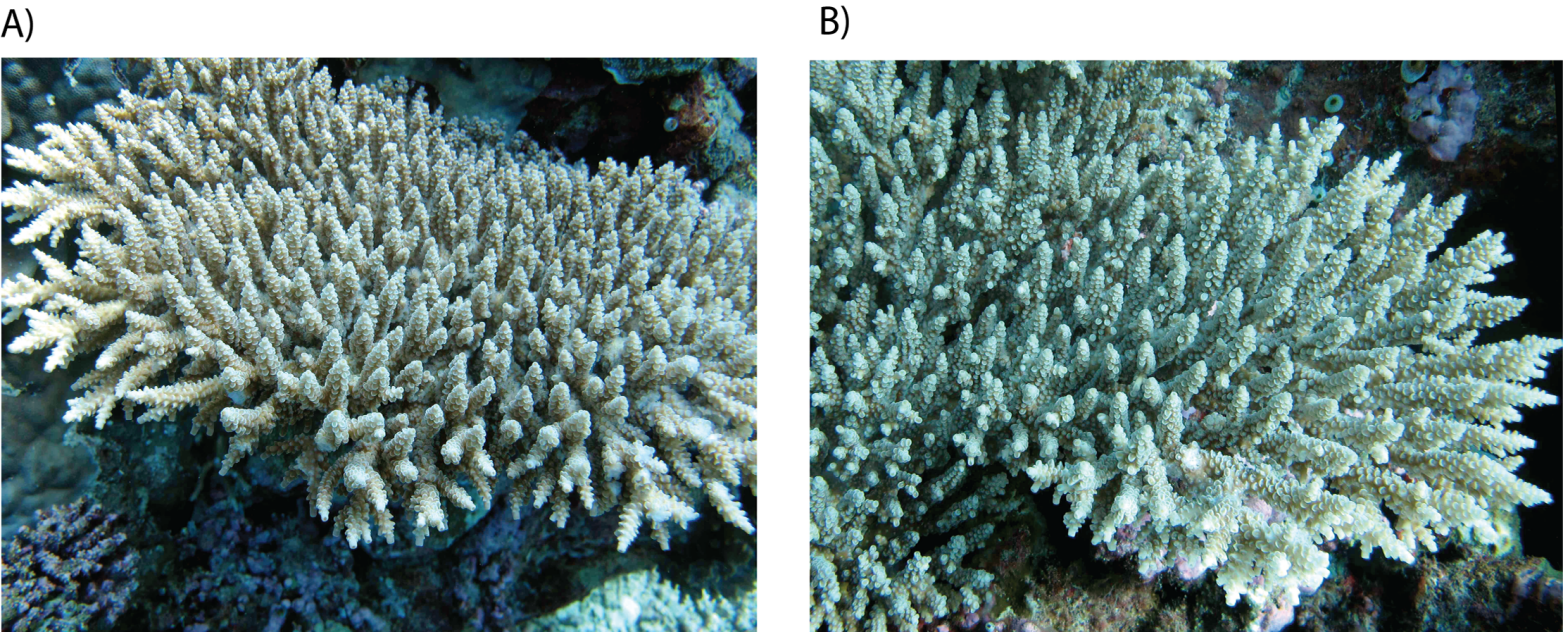
Colonies of the biannual spawning coral *Acropora tenuis* located < 10 m of each other within the same study site, but which spawned consistently in autumn (A) and spring (B).

Environmental conditions varied among sites depending on whether they were located at a sheltered lagoon, the inner- or outer-reef slope, or exposed to summer monsoonal storms from the west and more moderate winds and waves from the east in winter (S1 Fig). This level of environmental variation among sites did not influence the times of coral spawning by species or colonies; species that spawned during a single season did so during the same season at all sites, and for the biannual spawners a similar proportion of conspecific colonies spawned during both seasons at all sites. Indeed, at all sites conspecific colonies that spawned during autumn or spring were often located less than ten meters of each other (e.g. *Acropora tenuis*; Fig 3). Similarly, there was no evidence of *Symbiodinium* type in *A*. *tenuis* colonies influencing the season of spawning or the number of annual gametogenic cycles. All *A*. *tenuis* colonies that spawned in autumn and spring had identical *Symbiodinium* ITS2 DDGE profiles corresponding to type C40a (100% sequence similarity to *AY258485* [46]), which is a generalist symbiont of corals on Western Australian reefs [47].

### Microsatellite analysis of *A*. *tenuis* colonies

Two multilocus genotypes were shared among five *A*. *tenuis* colonies, but none of these occurred among colonies collected from the same sampling site. Both of these shared genotypes had a GP > 0.001, and therefore all individuals were included in the analysis. Between two and eight alleles were detected per locus in the two spawning groups along with high levels of heterozygosity in most loci (observed heterozygosity across all loci was 0.312 in the spring spawners 0.410 in the autumn spawners: (S1 Table). The autumn spawners had higher gene diversity as measured by expected heterozygosity (*H*_E_ = 0.519) despite having fewer alleles (*A* = 3.4 per locus) and less private alleles (*P*_VA_ = 2), compared with the spring spawners (expected *H*_E_ = 0.436, *A* = 5.2 and *P*_VA_ = 12) (S1 Table).

**Fig 4:**
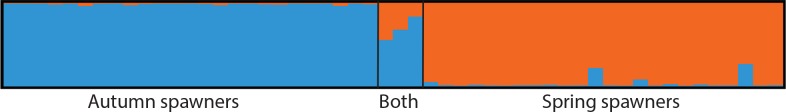
Assignment probabilities of *Acropora tenuis* colonies to the pre-defined autumn (blue) or spring (orange) cluster clusters (*K* = 2) using the LOCPRIOR model in STRUCTURE v2.3. Clumpak calculated this plot using 10/10 runs, a mean (LnProb) = -498.240, and similarity score = 0.971.

The initial analysis implemented in STRUCTURE without prior information on spawning season unambiguously identified two distinct clusters, with membership of colonies corresponding closely to their observed spawning season with *q* ≥ 0.75 in 40 out of 49 colonies (S2 Fig). The secondary clustering analyses that used the spawning season as prior information to assist clustering at *K* = 2 exhibited even stronger membership coefficients, with *q* > 0.90 in 47 of the 49 individuals that spawned in a single season (Fig 4). Two individuals that spawned in spring showed some affinity to the autumn cluster (average *q* to autumn cluster across ten runs was 0.22 and 0.27). The three individuals that spawned in both seasons also exhibited a degree of mixed ancestry, but all had stronger affinities with the autumn cluster with average *q* across the ten runs of 0.55, 0.68 and 0.84 (Fig 4). All replicate STRUCTURE runs in both analysis exhibited good convergence of the Markov Chain, as indicated by constant α and Ln likelihood within runs and highly congruent Ln P(D) across independent runs.

A major genetic divergence detected in the clustering analysis was supported by large estimates of differentiation between *A*. *tenuis* colonies spawning in different seasons (*F*_*ST*_ = 0.149and *G”*_*ST*_ = 0.469). Visual inspection of allele frequencies indicated that these differences were obvious at multiple loci (S1 Table), indicating that the observed patterns were not due to locus specific factors such as selection.

## Discussion

Biannual coral spawning is a persistent feature of the *Acropora* assemblages of the Scott Reef oceanic system. Our study demonstrates a primary mass spawning period during austral autumn and a smaller multi-specific spawning during spring, and adds to a growing number of studies documenting biannual coral spawning in north-western Australia (e.g. [48, 49]). There was no evidence of significant multi-specific spawning during other seasons or of individual colonies switching their primary season of spawning. Within seasons, spawning usually occurred in March (autumn) and October (spring), but can occur during the adjacent months or be ‘split’ over two consecutive months depending on the timing of the full moon, as on the Great Barrier Reef [1, 18].

The oceanic reefs of north-western Australia are in a region of transition between the equatorial reefs to the north, where spawning occurs over a protracted period but with a greater participation during the same discrete periods as at Scott Reef [1, 50], and the higher latitude reefs to the south, where there is little or no participation in a spring spawning [48, 51, 52]. Larval dispersal between the equatorial reefs and north-western Australia is facilitated by the South Java Current, the Indonesian Through Flow and the Holloway Current [23, 53–56]. The strongest southerly flow occurs during autumn months, coinciding with the predominant period of spawning throughout Western Australia. The strength and direction of current flow is weaker and more variable during spring and summer, with larval connectivity through the summer months probably reduced further by the frequency of monsoonal storms [54, 57]. The broad pattern of biannual spawning at the oceanic reefs of north-western Australia therefore reflects a genetic legacy of relative connectivity to the equatorial reefs [51] and the concentration of spawning activity during discrete seasons due to greater environmental variation at higher latitudes [1, 3, 11, 23]. The much reduced southerly flow during the spring and the lower energy reserves for oogenesis through the preceding winter months probably underlie the lower genetic diversity [58] and lack of spring spawning and on the higher latitude reefs of Western Australia. The existing evidence for tropical Western Australian reefs is that environmental conditions are suitable for coral gametogenesis during many months of the year, but spawning occurs during discrete seasons on norther reefs and becomes more protracted around a single season (autumn) with increased latitude [23, 48, 49, 51, 52].

The combination of reproductive, genetic and environmental data for Scott Reef further support the hypothesis that a colony’s season of spawning is primarily genetically determined, but that significant inter-annual variation in environmental conditions (e.g. sea water temperature, lunar phase) influence the month of spawning and the capacity to spawn twice a year. Most (98%) tagged colonies at Scott Reef spawned only once a year and consistently in the same season, which was evident in the prodigious genetic differentiation between the autumn and spring spawners of *A*. *tenuis*; the differentiation between colonies spawning during different seasons at Scott Reef was far higher (*F*_*ST*_ = 0.149, *G”*_*ST*_ = 0.469) than among colonies separated by 1000s of kilometres across Western Australia (*F*_ST_ = 0.067, *G”*_ST_ = 0.113; recalculated from [58]). Conversely, there was no evidence of environmental variation among sites across the Scott Reef system or of *Symbiodinium* partners influencing a colony’s spawning season or capacity to spawn more than once a year. A small proportion of colonies and species at Scott Reef had a second annual gametogenic cycle, as in other studies where individual corals have been resampled over multiple years [3, 8, 21, 30]. Favourable environmental conditions and food availability can produce multiple annual gametogenic cycles in corals [59] and at Scott Reef the second annual gametogenic cycle in colonies occurred in spring during the same year in different species, further suggesting that inter-annual variation in environmental conditions was responsible. In other years, these colonies spawned only during autumn and the three *A*. *tenuis* colonies had greater genetic affinities to the autumn spawners. There was, however, also evidence of mixed ancestry in a small proportion of *A*. *tenuis* colonies, including those with two annual gametogenic cycles. The autumn spawners exhibited higher genetic diversity but far fewer private alleles compared with the spring spawners, indicating not only that the autumn spawners have a larger effective population size, but also that gene flow is asymmetric, occurring primarily from the autumn to the spring spawners. The capacity for a second annual gametogenic cycle (during spring) may therefore exist only for the colonies whose primary season of spawning is autumn, whereas the spring spawners may not have the same capacity for a second cycle (during autumn) and to pass on newly mutated alleles. This hypothesis is based on evidence from a small sample size at Scott Reef, but is worthy of further investigation with more detailed reproductive and genetic data in coral assemblages where biannual spawning has been documented in both populations and individuals.

Temporal reproductive isolation and genetic differentiation between conspecific colonies in other studies of corals have suggested the presence of cryptic species [60–64]; for example, cryptic speciation within common *Pocillopora* lineages in north-eastern Australia [65–67]. Consistent spawning by conspecific colonies during discrete periods reduces morphological and genetic similarity, but a capacity for some colonies from the different reproductive groups to occasionally spawn during the same period may preclude speciation. Resolving the complex patterns of reproduction and genetic differentiation in coral species is confounded by the difficulty in identifying coral species based on morphology and the associated phenotypic plasticity [68–70], hybridization among species [71–73] and reproductive isolation within species [8, 60, 74, 75]. In this study, efforts were made to avoid errors in identification by focusing on colonies with morphology typical of their species. Of the colonies (*n* = 244) that were tagged and followed through time, 10% were excluded because their identities could not be confirmed following *in situ* and skeletal examination at the completion of the study, and differences in expert opinion also existed among other specimens. A detailed morphometric analysis was not conducted and for the many colonies in the random sample identifications were based only on *in situ* observations. Other studies of coral reproduction and genetics in the *Acropora* also rely on mostly *in situ* identification, with more detailed investigation for only a subset of type specimens invariably leading to some errors. Addressing these issues is logistically very difficult (see [17, 74]), but the requirement to do so depends on the research objectives. Species delineations may not be required if the aim of a study is to quantify the pattern of reproduction at the scale of the entire assemblage where data are obtained for genera and/or morphological groups (e.g. tabular *Acropora*), rather than individual species. Less detailed information is obtained, but the approach if far simpler and supports management actions to protect key periods of reproductive output from human pressures, while also ensuring that reproductive data for incorrectly identified species do not become entrenched in the literature. If, however, the goal of a study is to elucidate the species boundaries in coral assemblages, then detailed reproductive, morphometric and genetic analyses are clearly required over several years.

## Supporting Information

S1 Fig Variation in environmental conditions among sites through the study period. Current speed, chlorophyll concentrations, turbidity, and wave heights were quantified only at the Lagoon, Inner East and Inner West sites. Cover (%) of sand is and annual average for the period (2008–2010) of this study. Other parameter values are daily averages divided between summer and winter months, which accounted for the influence of monsoonal storms.
(PDF)

S2 Fig Clustering analysis of *Acropora tenuis* colonies with no prior information calculated in STRUCTURE v2.3. Upper panel shows Delta *K* as a function of *K*. Lower panel is the assignment probabilities of colonies at *K* = 2, with colonies that were identified in the reproductive surveys as autumn or spring spawners, and colonies that spawned in both seasons during one year. Values calculated in Clumpak using 10/10 runs, a mean (LnProb) = -501.5, and similarity score = 0.999.
(PDF)

S1 Table Details of the five microsatellite markers in *Acropora tenuis* colonies that spawned in autumn or spring. Given are the sample sizes (in brackets after season name), number of alleles (*A*), the proportion of expected (*H*_*E*_) heterozygotes, and the fixation index (*F*_IS_) calculated for each locus and averaged across loci (All _loci_) for each reproductive season, and the number of private alleles (*P*_*V*_*A*) for each reproductive season. Bolded *F*_IS_ estimates indicate significance at p<0.05 after sequential bonferroni correction.
(PDF)

S1 Text Variation in habitat conditions among sites. (PDF)
