# Supplementary figures and images for: Biannual Spawning and Temporal Reproductive Isolation in Acropora Corals
Source: PLoS One. 2016 Mar 10;11(3):e0150916. doi: 10.1371/journal.pone.0150916 (PMC4786224; doi:10.1371/journal.pone.0150916)

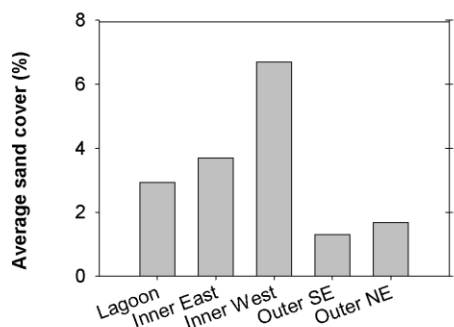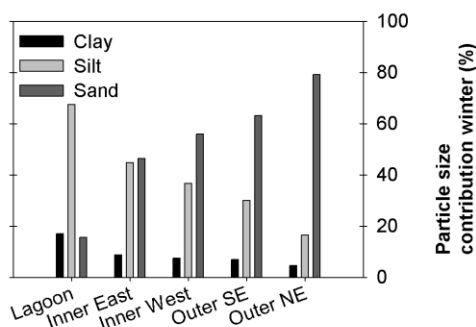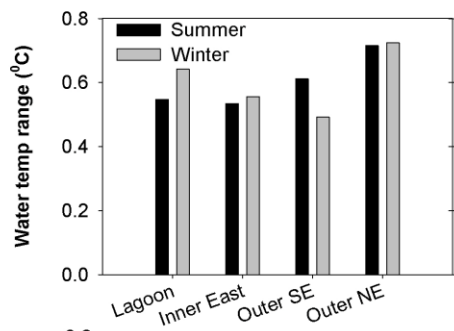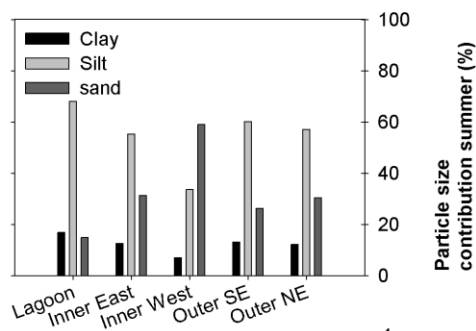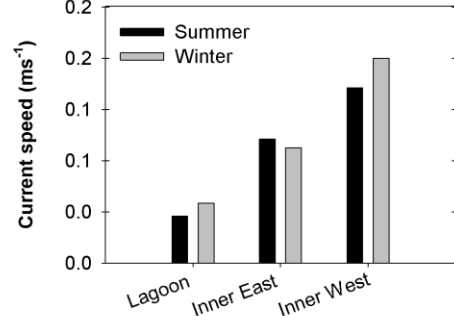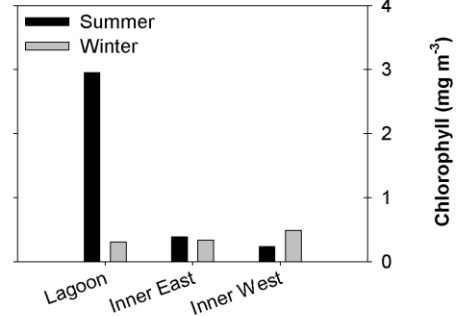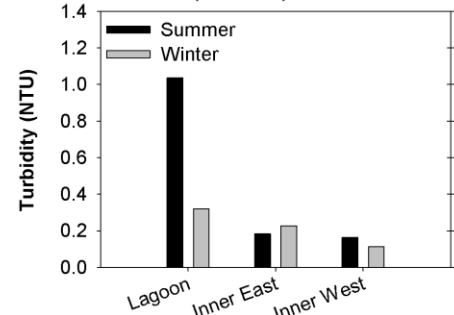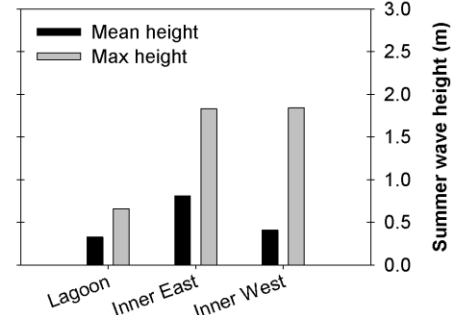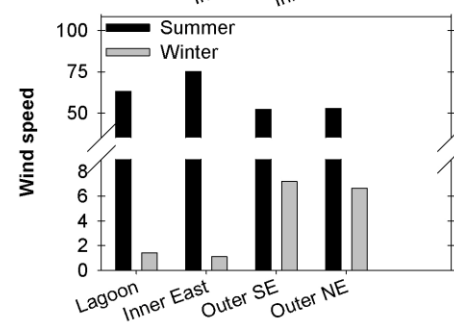

Supplement: S1 Fig — Current speed, chlorophyll concentrations, turbidity, and wave heights were quantified only at the Lagoon, Inner East and Inner West sites. Cover (%) of sand is and annual average for the period (2008–2010) of this study. Other parameter values are daily averages divided between summer and winter months, which accounted for the influence of monsoonal storms. (PDF) [file pone.0150916.s001.pdf]

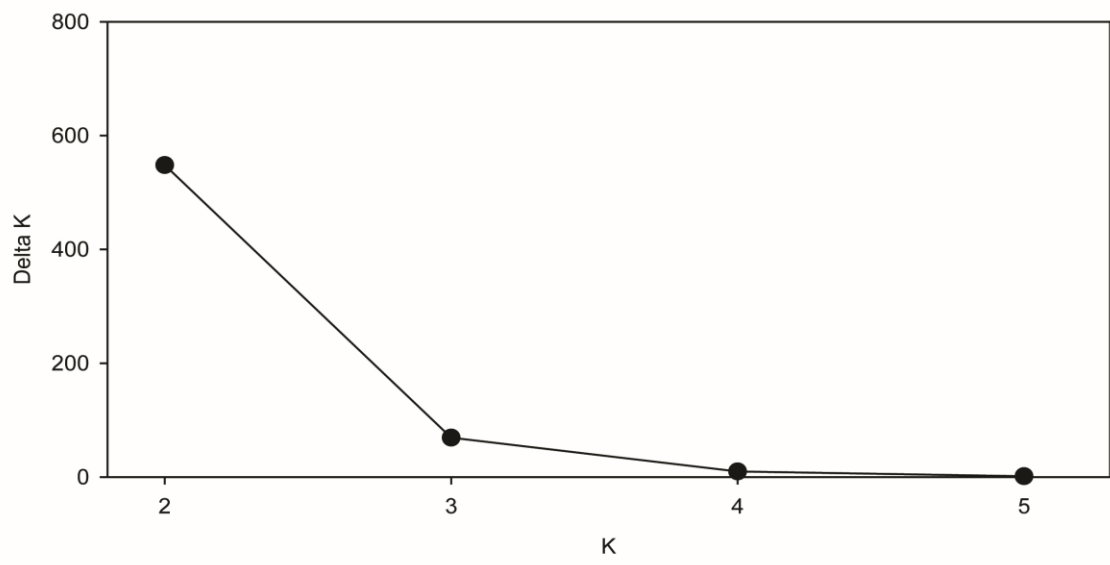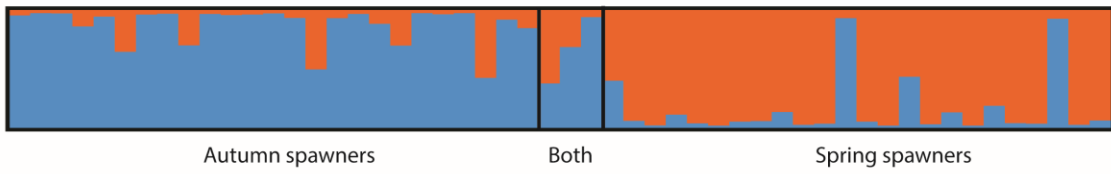

Supplement: S2 Fig — Upper panel shows Delta K as a function of K. Lower panel is the assignment probabilities of colonies at K = 2, with colonies that were identified in the reproductive surveys as autumn or spring spawners, and colonies that spawned in both seasons during one year. Values calculated in Clumpak using 10/10 runs, a mean (LnProb) = -501.5, and similarity score = 0.999. (PDF) [file pone.0150916.s002.pdf]
