# Supplementary material for: Biannual Spawning and Temporal Reproductive Isolation in Acropora Corals
Source: PLoS One. 2016 Mar 10;11(3):e0150916. doi: 10.1371/journal.pone.0150916 (PMC4786224; doi:10.1371/journal.pone.0150916)
Supplement: S1 Table — Given are the sample sizes (in brackets after season name), number of alleles (A), the proportion of expected (HE) heterozygotes, and the fixation index (FIS) calculated for each locus and averaged across loci (All loci) for each reproductive season, and the number of private alleles (PVA) for each reproductive season. Bolded FIS estimates indicate significance at p<0.05 after sequential bonferroni correction. (PDF) [file pone.0150916.s003.pdf]

Spawning season		*Acr_53*	*Amil2_10*	*Amil2_011*	*Amil2_ 012*	*Amil2_018*	*All $_{loci}$*	P_VA
**Autumn (n=25)**	A	3.0	3.0	4.0	3.0	4.0	3.4	2.0
	H_O	0.542	0.174	0.440	0.440	0.455	0.410	
	H_E	0.494	0.419	0.657	0.503	0.524	0.519	
	F_{IS}	-0.097	**0.585**	**0.330**	0.126	0.132	**0.215**	
**Spring (n=24)**	A	3.0	8.0	6.0	5.0	4.0	5.2	12.0
	H_O	0.125	0.591	0.182	0.375	0.286	0.312	
	H_E	0.119	0.549	0.419	0.576	0.516	0.436	
	F_{IS}	-0.051	-0.077	**0.567**	0.349	**0.446**	**0.247**	
