# Supplementary material for: Biannual Spawning and Temporal Reproductive Isolation in Acropora Corals
Source: PLoS One. 2016 Mar 10;11(3):e0150916. doi: 10.1371/journal.pone.0150916 (PMC4786224; doi:10.1371/journal.pone.0150916)
Supplement: S1 Text — (PDF) [file pone.0150916.s004.pdf]

The contribution of parameter statistics to the differences in physical conditions across the reef system was explored using Principal Components Analysis (PCA) of normalised data and the BEST analysis in the software PRIMER [1]. Of the 19 parameters initially investigated, nine remained after excluding those which explained a low proportion of variation and those highly correlated ($r > 0.9$) with another. The final parameters were: percentage cover of sand, cumulative wind speeds, range in water temperature, rate and composition of sedimentation, turbidity and chlorophyll concentration (fluorescence), and maximum current speed and wave height. For all parameters, (but cover of sand) daily averages were calculated and divided between summer and winter months, to account for the influence of monsoonal conditions.

Sedimentation rates and particle sizes at each site were quantified using replicate (n = 5) sediment traps of 700 mm length and 110 mm diameter, with an internal baffle system to exclude large organisms. Traps were exchanged at intervals of between 3 to 6 months. The sediment contained within each trap was wet sieved through 500 µm, frozen and transported to the Particle Analysis Service Laboratory of CSIRO where samples were processed to determine particle size distributions (PSD) and the total dry weight of sediment. The percentage cover of sand at each site was quantified along permanent transects (250 m) during the study period (2008, 2010) according to the AIMS LTM methods [2]. Wind data were obtained from the Australian Bureau of Meteorology weather station at Browse Island, approximately 160 km to the east of Scott Reef. Wind speeds were measured as km h-1 in 22.5 degree increments, at hourly intervals from 1990 to 2009. The remaining physical parameters were quantified from October 2008 to June 2010 using instrument arrays at or adjacent to each site, which consisted of Seabird SBE16 loggers (www.seabird.com) with integrated Wetlabs ECO FLNTU and ECO PAR optical sensors (www.wetlabs.com).

1. Clarke KR, Warwick RM. Change in Marine Communities: An Approach to Statistical Analysis and Interpretation (2nd ed.): Plymouth, U.K.; 2001.
2. Jonker M, Burgess SC, Coleman G, Osborne K. Long-term monitoring of the Great Barrier Reef. Townsville: Australian Institute of Marine Science, 2007.
